# Laparoscopic vs. Robotic Gastrectomy in Patients with Situs Inversus Totalis: A Systematic Review

**DOI:** 10.1155/2023/3894561

**Published:** 2023-03-02

**Authors:** Anmol Multani, Simran Parmar, Elijah Dixon

**Affiliations:** ^1^Kansas City University – College of Medicine, Joplin, MO, USA; ^2^University of Calgary, Calgary, AB, Canada

## Abstract

**Background:**

Situs inversus totalis (SIT) is a rare genetic anomaly involving the mirror-image transposition of organs. This transposition can potentially make surgical treatments difficult because of the reversed anatomy and intraoperative confusion. The aim of this systematic review is to compare the perioperative outcomes and safety of robotic and laparoscopic gastrectomy in patients with SIT.

**Methods:**

We included full-text case reports with brief reviews and standalone case studies on SIT patients age ≥21, undergoing laparoscopic or robotic gastrectomy. We excluded case studies focusing on procedures other than laparoscopic and robotic gastrectomy, namely, open gastrectomy, gastric banding, and gastric bypass. English was selected as the language and articles published in the last 10 years were selected with a date range from Jan, 2011, to Aug, 2021. We focused on intraoperative and postoperative outcomes including blood loss, vascular aberrancy, operation duration, mortality, operative complications, duration of hospitalization, and follow-up interval. Online databases included Clinical Key, Embase, ScienceDirect, Ovid, and Google Scholar. The last search was conducted on Aug 15, 2021. For all eligible articles, risk of bias assessment was carried out using JBI critical appraisal checklist (Table 1). Continuous data were analyzed using *t*-test with *p* value of 0.05.

**Results:**

From our search, we retained 29 case reports which reported information from 30 cases. The results reported in each study were summarized (Table 2). The laparoscopic procedure was used in 21 cases and robot-assisted surgery was used in 9 cases. Operative time was mentioned in 24 out of the 30 cases and the average operative time was 205.67 min. Blood loss was reported in 16 out of the 30 cases, with an average blood loss of 51.9 mL. Hospital stay information was provided in 26 out of the 30 cases, with an average length of stay of 8.5 days. A statistically significant difference was not found for the operative time, length of hospitalization, or age of the patient. However, intraoperative blood loss in robot-assisted gastrectomy was lower compared to laparoscopic gastrectomy, with a *p* value of 0.0293. Perioperative death was not reported in any of the cases. Only three cases of postoperative complications were reported in laparoscopic surgery. Only one of the three cases suggested that the complication was due to an anomaly, whereas the other two of them reported complications due to procedural errors.

**Conclusion:**

Laparoscopic and robotic gastrectomy can be safely used for SIT patients if performed cautiously. Some precautions include thoroughly assessing anatomical aberrations using preoperative imaging, adjusting the operative set up, and having experienced surgeons. The robotic approach may have a few advantages over laparoscopic procedures that may enhance the surgical safety for SIT patients and need to be further explored in future research. Advantages of the robotic approach may include improved surgical safety with better visualization of the surgical field, promoting the stability of surgical instruments and perhaps allowing ease of surgical orientation and positioning when operating on patients with SIT. Further research in this field is merited.

## 1. Introduction

Situs inversus totalis (SIT) is a congenital disorder in which the thoracic and abdominal organs have reversed positions, such as a mirror-image of the normal anatomy [1]. It is a rare genetic condition with multiple mutations and an incidence rate of 1 in 5000 to 20000 births [1]. The unusual anatomy of patients with SIT and the lack of a standardized surgical approach poses technical challenges during the diagnostic process and its surgical management.

Morbid obesity is currently on the rise in patients with and without SIT, and bariatric surgery has been effective in helping patients attain sustained weight loss. The most common form of bariatric surgery is gastrectomy. The prevalence of morbid or clinically severe obesity rose from 4.7% to 9.2% in the United States in the last two decades [2] and is contributing toward the risk of chronic conditions such as cardiovascular disease, hypertension, diabetes mellitus, and certain malignancies [3]. There is a growing prevalence of obesity and the impact of bariatric procedures such as gastrectomy on patients. The challenges posed during gastrectomy procedures by the anatomic variations in SIT patients warrant an investigation into the safety of these surgical procedures.

Gastrectomy is not only indicated for managing weight loss but for other pathologies as well such as gastric neoplasm, ischemia, ulcers, and bleeding in patients with SIT. Gastrectomy allows the surgical resection of a gastric neoplasm and prevents disease progression. Gastric cancer is the third leading cause of cancer-related death worldwide [4]. Reversed anatomy in SIT patients increases the operative complexity of surgical procedures such as gastrectomy. Intraabdominal malignancies can occur in patients with SIT, warranting foresight on the surgical approach to mitigate complications.

Gastrectomy can be performed either laparoscopically or robotically in SIT patients with the goal of treating obesity and gastric cancer. No published reviews have compared laparoscopic and robotic gastrectomy to date, signifying the need to analyze the literature on this topic and help surgeons choose and provide the best surgical treatment to SIT patients. The aim of this systematic review is to compare laparoscopic and robotic gastrectomy in the safety and operative outcomes, such as operative time, blood loss, hospital stay length, mortality, and morbidity, for SIT patients, who pose technical operative challenges due to the reversed position of their intraabdominal organs. We will also highlight recommendations to overcome the technical challenges of SIT in gastrectomy to enhance the quality of surgical care available to SIT patients.

## 2. Methods

In the present systematic review, we followed the Preferred Reporting Items for Systematic Reviews and Meta-Analyses literature search extension (PRISMA-S) guidelines wherever possible [5, 6].

### 2.1. Types of Studies

We included published full-text case reports with brief literature reviews and standalone case studies of SIT patients undergoing gastrectomy. We excluded cases studying situs inversus partialis instead of situs inversus totalis. Review articles and cases reported in video format were also excluded.

### 2.2. Population

SIT patients undergoing gastrectomy were classified as adults with age ≥21. There was no upper limit used on the age of the patients. Data included patients of both sexes. Patient data were not stratified based on comorbidities or BMI.

### 2.3. Intervention

We initially considered all gastrectomy procedures in SIT patients. We then further narrowed the search results using the search terms “robotic gastrectomy” and “laparoscopic gastrectomy.” We excluded case studies focusing on procedures other than laparoscopic and robotic gastrectomy, namely, open gastrectomy, gastric banding, and gastric bypass. Articles published in the English language were selected. Case studies published within the last ten years (Jan, 2011, to Aug, 2021) were selected, and articles were excluded because they were published outside of the selected range of publication date. We excluded articles published more than ten years ago to ensure we did not analyze information from outdated case studies. Additionally, by including studies published from 2011 onward, we were able to include information from the first robotic gastrectomy in SIT patients, which was performed in 2012.

### 2.4. Outcomes

We included case studies reporting specific types of gastrectomy procedures, intraoperative outcomes (blood loss, vascular variation, and operation duration), and postoperative outcomes (mortality, complications, hospital stay duration, and follow-up interval). We focused on postoperative mortality and morbidity on a short-term and long-term basis, with follow-up intervals of studies ranging from a minimum of 24 hours to a maximum of 4 years.

Two articles were excluded because they did not report the outcomes of interest.

#### 2.4.1. Information Sources

A comprehensive online database search was conducted independently by two reviewers (AM, SP) till August 15, 2021, using multiple medical databases, including PubMed, Clinical Key, Embase, ScienceDirect, Ovid, and Google Scholar.

#### 2.4.2. Search Strategy

To search for case studies, we used these descriptors in PubMed: “case study” (All Fields) OR “case report” (All Fields). After some rounds of trial and refinement of search terms, we formulated the final search term for PubMed as follows: (gastrectomy OR laparoscopic gastrectomy OR sleeve gastrectomy OR distal gastrectomy OR proximal gastrectomy OR subtotal gastrectomy OR robotic gastrectomy OR robotic-assisted gastrectomy OR robot-assisted gastrectomy or gastric resection) AND (situs inversus OR Kartagener syndrome OR situs inversus totalis) AND (case study OR case report OR literature review). The publication date was limited to ten years (2011–2021), and the language was limited to English. Since the research for this topic is limited, we did not include outcome terms (mortality, safety, and postoperative complications) in the search term to capture more reports and data.

The search term created in PubMed was customized based on the specific characteristics of each database. For Google Scholar, we used the following advanced search:

1. With all of the words: gastrectomy AND situs inversus
2. With at least one of the words: laparoscopic gastrectomy OR sleeve gastrectomy OR distal gastrectomy OR proximal gastrectomy OR subtotal gastrectomy OR robotic gastrectomy OR robotic-assisted gastrectomy OR robot-assisted gastrectomy or gastric resection AND (situs inversus OR Kartagener syndrome OR situs inversus totalis)
3. Where my words occur in the title of the article

For Clinical Key, we filtered articles by selecting journal as the source type and full text and MEDLINE option. Using the same search terms previously used for PubMed, we selected articles published in the last ten years. Similarly, while using ScienceDirect and Embase, we used the same search terms and filtered the results by selecting case reports and review articles as article types published since 2011. The aforementioned search terms were also searched via OvidSP using Ovid MEDLINE and In-Process and Other Non-Indexed Citations. The database coverage was from Jan, 2011, to Aug, 2021.

Finally, all records were collected into one Mendeley library in order to delete duplicates. References remaining after this step were exported to an excel file with essential information for screening, including authors' names, publication year, journal, DOI, URL link, and abstract.

It was not necessary to use any internal or externally derived machine learning classifier for the initial search, and further elimination of ineligible and duplicated reports resulted in a limited data set of 46 papers.

### 2.5. Eligibility Criteria

Articles of this review were chosen using the PICOS elements, where population [P] = Situs inversus totalis patients, intervention [I] = different laparoscopic and robotic gastrectomy procedures, including laparoscopic sleeve, distal, proximal, subtotal or distal subtotal gastrectomy and robotic-assisted distal, total, and proximal or radical subtotal gastrectomy. For comparison [C] = patients without SIT, outcome [O] = intraoperative outcomes (blood loss, operation duration, and mortality) and postoperative outcomes (mortality, complications, morbidity, vascular variations, hospital stay duration, and follow-up interval), and study design [S] = published standalone case reports and case reports with brief reviews.

#### 2.5.1. Selection Process

Duplicate checking of all records was performed independently by two reviewers (AM and SP) using titles and abstracts. We retrieved full-text copies of potentially relevant articles to carry out the full-text assessment. Inconsistencies found by the two reviewers were discussed in research meetings held with the primary investigator to resolve the inconsistences and reach a consensus. In case of disagreement, full-text was screened for inclusion independently by two researchers and later discussed with the primary investigator. The final list of eligible articles was confirmed with discussion and consensus amongst the authors (AM, SP, and ED).

#### 2.5.2. Data Extraction and Management

Two researchers (AM and SP) collected information from eligible articles using a data extraction sheet in Excel. Information extracted was compared and discrepancies were resolved through discussion. AM entered data into the extraction form and SP double-checked for accuracy. Information regarding the endpoints was clear, and it was not necessary to contact the authors of the case reports providing further details. We extracted the data using the following key study features where possible:

1. Study characteristics–risk of bias, study design, source of publication, type of study, author, and year
2. Patient characteristics–age, sex, anatomical variations (vascular variations) that may have posed surgical challenges
3. Surgery characteristics–type of intervention, surgeon and patient position, and surgeon hand used (right, left or both)
4. Intraoperative outcomes–blood loss, operation duration, vascular variation, and mortality
5. Postoperative outcomes–mortality, complications, hospital stay duration, and follow-up interval

Results were compatible with each outcome domain. No changes were made to the process used to select results within eligible outcomes and domains. No changes were made to the inclusion and exclusion criteria for the domains.

#### 2.5.3. Assessment of Risk of Bias in Included Studies

Two authors (AM and SP) independently assessed the risk of bias in included studies, and one author acted as arbitrator (ED). To assess for risk of bias for case reports belonging to the descriptive studies category, we used the Joanna Briggs Institute (JBI) critical appraisal checklist for case reports (last amended in 2017) (Table 1). Each article was assessed using eight questions by selecting answers “yes,” “unclear,” “no,” or “not applicable.” Articles were evaluated using the criteria: low risk of bias–more than 70% “yes” score, moderate risk of bias – 50% to 69% “yes” score, and high risk of bias–less than 49% “yes” score. Two authors independently applied this tool to each case report to reach an overall appraisal judgment with supporting justifications for each article (Table 2).

#### 2.5.4. Synthesis of Results

We grouped studies based on the type of gastrectomy performed (laparoscopic vs. robotic) to determine if the clinical outcomes differed between them. The continuous variables were reported as standard means with standard deviation, and categorical variables were reported as the frequency with respective proportion (percentage). *R* statistical software was used to run statistical tests and provide summary statistics, including mean, median, and range. We used mean difference as the effect measure along with the associated 95% confidence intervals. We also conducted Student's *t*-test to determine the significance of the differences. A *p* < 0.05 was considered significant.

Using outcome domains and results reported in each study, we created a summary of findings in Table 3. Since all studies scored low on the risk of bias assessment, outcome results are ordered by publication date rather than the risk of bias to reveal patterns in the data.

We did not conduct a subgroup analysis of the case studies as there were insufficient studies sharing the same type of procedure and patient demographics. While examining the effect of study quality on the magnitude and direction of the effect, studies were found to have a low risk of bias overall in the sensitivity analysis.

## 3. Results

**Figure 1:**
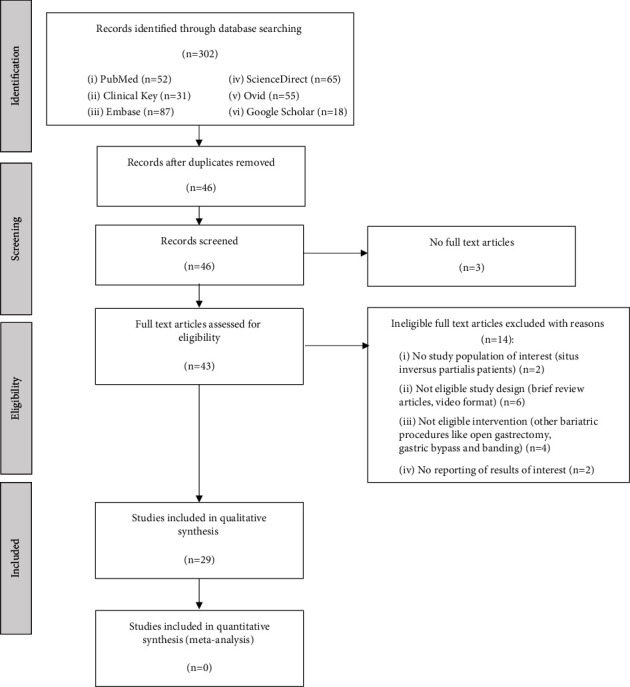
Flowchart showing search strategy and selection of retained articles for analysis.

The flow of studies through the systematic review process is shown in Figure 1. A search was run in electronic databases between August 10 and August 15, 2021. Through the search, we retrieved 302 records, including duplicates. No other records were added from other sources, such as reference sets. Duplicates were removed, yielding 46 title-abstract records. After eliminating three articles with unavailable full-text versions, we screened 43 full-text articles. During the full-text screening stage, we excluded 14 articles and assessed 29 articles that were eligible according to the inclusion criteria. There were 20 laparoscopic gastrectomy cases and 9 robotic gastrectomy cases. Data extracted from the eligible articles for the review are categorized under different domains, and the main outcomes of interest are listed in Table 3.

### 3.1. Risk of Bias Assessment

We used the JBI critical appraisal checklist listed in Table 1 for each of the included studies. A summary of the assessments is provided in Table 2. All studies were rated as low on the overall risk of bias.

### 3.2. Different Surgical Procedures

Distal gastrectomy was the most common among the gastrectomy procedures performed with a laparoscopic or robotic approach (Table 4).

### 3.3. Operative Time, Blood Loss, and Hospital Stay

Operative time was mentioned in 24 out of the 30 cases. The median operative time was 202.5 min with an average of 205.67 min. The laparoscopic procedure was used in 21 cases with a median operative time of 200 min. Robotic surgery was used in 9 cases, and the median operative time was 205 min. Blood loss was reported in 16 out of 30 cases, with a median of 47.5 mL and an average of 51.875 mL. Hospital stay information was provided in 26 out of the 30 cases. The median hospital stay was 8 days, and the average was 8.538 days.

Bivariate analysis was performed to compare the operative time, age, blood loss, and hospital stay for the two types of procedures (Table 5). A statistically significant difference was not found between operative time, age of the patient, and hospital stay length. However, intraoperative blood loss in the laparoscopic procedure (63.50 ± 36.21 ml) had a statistically significant difference compared to robot-assisted gastrectomy (32.50 ± 12.54 ml), with the *p* value of 0.0293. The robot-assisted procedure caused less bleeding (32.50 mL).

### 3.4. Mortality

No cases of postoperative death were reported.

### 3.5. Complication

A total of three cases reported postoperative complications. All three cases were linked to laparoscopic surgery. Only one of the cases suggested that the complication was due to an anomaly associated with SIT condition [7]. The other two articles reported complications as a result of procedural errors rather than in relation to SIT [8, 9]. The altered connection of lymphatic vessels is one such rare anomaly in SIT. The surgical procedure disrupted small lymphatic vessels that had a varying connection with the thoracic duct, causing chylothorax and pericardial effusion as a postoperative complication [7]. Another reported complication was a postoperative mechanical obstruction due to adhesion, requiring a take back to the operating theatre. This was identified as an error of the procedure and not linked to the situs inversus condition [9]. One of the other reports mentioned postoperative leaks in the gastrosplenic ligament as a complication [8]. This was not due to the altered anatomy in SIT patients, but because of the previous gastric banding procedure and adhesions, making the dissection difficult.

### 3.6. Anatomic Variations Associated with SIT

Some of the cases reported anatomic alterations accompanying the condition of situs inversus totalis, which made surgical resections longer and more challenging. Vascular anomalies are the most common form of anomalies found in SIT patients and have been described in five cases (Table 6). A lymphatic anomaly was also noted where diaphragmatic lymphatic vessels joined thoracic ducts. Cardiovascular and pulmonary anomalies in SIT patients were reported in the form of an atrial septal defect [10] and respiratory insufficiency due to the Kartagener syndrome in SIT patients [11, 12].

## 4. Discussion

Robot-assisted gastrectomy has been favored over laparoscopic gastrectomy due to multiple factors. It is less invasive, offers better visual field exposure, promotes the stability of instruments [13], and allows faster sewing [14]. Unlike robotic surgery, laparoscopic gastrectomy involves a higher degree of cooperation between the operator and the 1^st^ assistant and more chances of communication error [13]. As per the analysis of existing literature, robotic gastrectomy causes significantly less intraoperative blood loss (*p* = 0.0293); however, it is similar in the operative time and hospital stay. Additionally, all the surgical complications mentioned in the literature were only in laparoscopic surgery. Robotic surgery also does not require reversal of the surgeon's position or monitor, which can mitigate confusion and technical issues [15, 16]. Surgeons can operate from their standard position because robotic arms are flexible [14] and allow instruments to be handled similarly from either side if robotically placed [17]. They can also continue to use their dominant hand by staying in the standard position, which can avoid confusion [16, 18]. However, both forms of surgery require a thorough investigation of the position of the major organs to anticipate the complexity of SIT [19, 20] and identify any vascular anomalies and variations in branching patterns preoperatively [15, 18, 20]. It is also recommended that surgeons operating on SIT patients be experienced and have advanced skills, regardless of the method of surgery chosen [14]. SIT's reversed anatomy makes the procedure more complex and resection more difficult, especially since it requires a precise and careful approach, and it may conflict with the commonly used surgical practices [13]. Overall, robotic gastrectomy is considered a safe procedure in SIT patients and has similar outcomes to patients without SIT [21].

Based on the case reports showing no mortality and low morbidity, it can be concluded that gastrectomy procedures performed on SIT patients, either laparoscopically or robotically, are safe and successful. The condition of SIT, however, may enhance the time and the level of complexity of the procedure [22]. As with any form of surgery, gastrectomy may cause postoperative complications. Only five of the 30 currently published case reports noted complications, mostly due to procedural errors and not due to the SIT condition.

Before performing invasive procedures such as surgery, the surgical team must establish SIT diagnosis preoperatively as the patient may be unaware of the diagnosis. This can be carried out by viewing reversed positions of thoracic and abdominal organs through multiple imaging modalities, including chest radiography and ultrasound [1] and abdominal CT and ultrasound [23]. Preoperative diagnosis can prevent situations where an intraoperative revelation of SIT occurs, and more time is spent adapting to the reversed anatomical position [1, 24]. It also allows reevaluation of the operative setting relative to the reversed anatomy [25] and preparation for technical challenges.

The studies emphasized the importance of preoperative anatomic assessment as SIT is associated with a ten times greater risk of cardiovascular anomalies [26]. Vascular aberrations have been commonly found in patients with SIT. Preoperative imaging, such as 3D reconstructive CT angiography, can be used to identify anatomical variations in arteries and veins [22, 25–29] and ascertain anatomy that can cause further complications [25]. Careful assessment of vascular anomalies can help prevent complications such as injury to the blood vessels, bleeding, and pancreatic injury [29–31].

To reduce the risk of complications, OTs can be arranged in a way that surgeons can operate more ergonomically [25, 26], and surgical teams can be briefed beforehand to ensure proper orientation and reduce operative time due to adaptation difficulties [1]. A preoperative discussion with the team members is also crucial for coordination between the operators and assistants [11, 32]. Port placement and assistant and surgeon positions can also be planned preoperatively to ensure that the operator is comfortable and acquainted [8, 31].

Vast surgical experience in bariatric laparoscopy with good eye-hand coordination, meticulous skills and knowledge of anatomy, and the varying vascular branching patterns can help reduce negative surgical outcomes [8, 10, 13, 33]. Experienced surgeons may have an easier transition in orienting themselves according to the altered anatomy [1, 26, 27].

There is currently no consensus on the side from which a surgeon should operate in laparoscopic surgery of SIT patients. To avoid confusion created by the altered anatomy of SIT patients, some surgeons reversed their positions and port placement to allow for adaptation to the mirror-image anatomy [9, 22, 23, 25, 28, 30, 31], while others retained the standard positions and port placement [10, 11, 27, 32]. If standing on the opposite side, the surgeon should be able to use their nondominant hand sufficiently well to handle instruments [9, 25, 26, 29]. Left-handed surgeons may be at an advantage when operating from the opposite side [28]. However, other studies suggested not reversing positions and using standard positions to avoid confusion [27, 32]. To preserve the same surgeon position and instrument placement, a method involving a single incision through the umbilicus can be used instead of having multiple ports at different locations [12]. Another method would be to operate from the standard side while using two monitors, where a second monitor would show reversed projection images of the organs during important dissections [23].

There were a few limitations in this review. Our analysis of published case reports cannot be used to make conclusions about causal relationships. Since the case studies were selected to be analyzed, there is a risk of selection bias. Not all the studies included relevant information and data, which may affect the statistical results. The inclusion of a scant number of studies published on robotic gastrectomy is another limitation of this review. Using articles only published in the English language can be another limitation. The inclusion of studies only published in the last ten years can be a strength as it increases the relevance of the results to current clinical care. Additionally, the systematic and thorough search through the various databases is a strength.

## 5. Conclusion

We assessed the implications of SIT on the outcomes of laparoscopic- and robot-assisted gastrectomy and found that both surgical interventions are safe. Preoperative diagnosis of SIT, identification of anatomical alterations, and intraoperative caution by experienced surgeons enhance the safety of these procedures. Robotic gastrectomy appears to have advantages over laparoscopic gastrectomy, including less blood loss, more flexibility, and fewer errors due to the change in the surgeon's position or lack of coordination. Further research in this field is merited.

## Figures and Tables

**Table 1:** The Joanna Briggs Institute (JBI) critical appraisal checklist for case reports.

(1) Were patient's demographic characteristics clearly described?	Yes	No	Unclear	Not applicable
(2) Was the patient's history clearly described and presented as a timeline?	Yes	No	Unclear	Not applicable
(3) Was the current clinical condition of the patient on presentation clearly described?	Yes	No	Unclear	Not applicable
(4) Were diagnostic tests or assessment methods results clearly described?	Yes	No	Unclear	Not applicable
(5) Was the intervention(s) or treatment procedure(s) clearly described?	Yes	No	Unclear	Not applicable
(6) Was the postintervention clinical condition clearly described?	Yes	No	Unclear	Not applicable
(7) Were adverse events (harms) or unanticipated events identified and described?	Yes	No	Unclear	Not applicable
(8) Does the case report provide takeaway lessons?	Yes	No	Unclear	Not applicable

Overall appraisal: Include □ exclude □ seek further info □.

**Table 2:** Risk of bias assessment according to the JBI critical appraisal checklist.

Author	JBI Q1	JBI Q2	JBI Q3	JBI Q4	JBI Q5	JBI Q6	JBI Q7	JBI Q8	Bias risk
Namikawa T., Maeda M., Yokota K., et al.	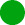	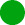	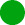	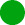	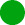	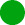	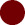	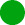	Low
Sivakumar J., Crosthwaite G.	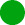	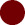	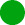	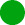	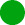	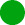	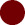	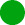	Low
Abbey E., Yang F., Qi L., et al.	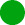	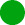	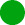	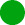	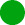	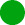	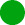	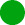	Low
Takeno A., Masuzawa T., Katsuyama S., et al.	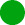	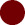	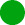	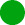	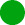	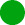	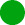	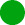	Low
Bawahab M. A.	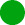	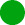	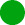	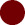	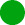	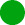	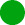	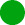	Low
Yoshimoto T., Yoshikawa K., Tokunaga T., et al.	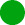	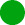	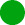	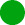	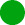	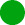	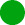	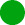	Low
Ojima T., Nakamura M., Nakamori M., Yamaue H.	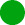	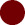	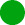	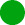	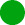	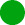	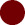 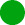	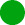	Low
Shibata K., Kawamura H., Ichikawa N., et al.	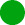	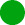	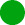	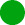	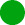	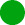		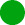	Low
Zhou, Lan et al.	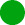	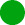	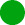	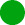	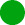	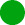	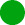	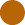	Low
Dai H. bin, Wang Z. C., Feng X. B., et al.	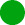	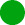	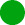	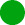	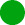	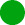	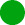	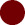	Low
Villalvazo Y., Jensen C. M.	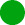	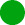	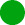	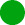	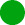	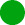	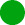	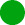	Low
Gundes, Cetin et al.	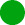	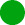	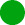	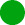	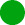	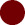	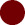	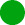	Low
Suh B. J.	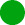	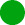	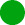	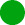	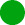	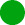	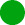	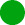	Low
Cao Y., Li J., Shen L., et al.	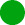	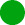	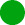	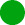	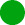	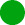	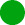	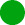	Low
Aziret, Karaman et al.	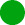	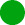	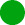	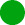	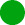	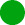	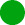	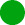	Low
Alhossaini R., Hyung WJ.	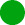	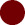	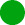	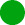	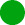	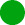	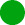	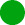	Low
Kigasawa Y., Takeuchi H., Kawakubo H., et al.	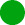	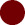	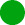	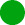	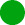	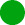	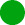	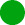	Low
Yazar F. M., Emre A., Akbulut S., et al.	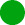	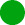	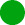	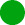	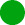	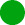	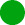	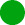	Low
Morimoto M., Hayakawa T., Kitagami H., et al.	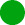	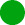	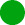	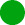	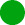	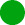	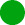	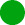	Low
Ye M. F., Tao F., Xu G. G., Sun A. J.	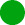	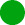	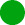	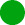	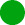	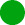	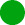	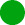	Low
Genser, Tayer et al.	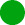	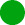	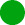	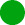	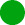	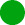	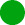	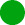	Low
Zhu H., Yang K., Hu J. K.	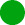	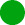	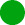	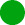	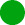	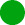	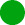	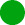	Low
Stier C., El-sayes I., Theodoridou S.	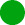	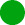	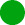	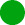	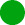	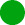	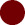	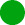	Low
Sumi Y., Maehara R., Matsuda Y., et al.	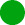	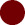	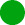	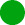	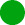	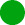	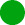	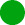	Low
Min S. H., Lee C. M., Jung H. J., et al.	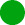	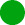	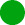	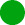	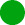	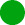	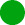	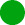	Low
Fujikawa H., Yoshikawa T., Aoyama T., et al.	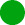	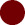	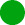	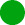	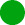	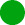	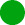	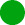	Low
Deutsch G., Gunabushanam V., Mishra N., et al.	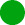	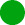	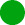	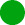	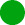	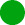	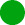	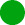	Low
Kim H. B., Lee J. H., Park D. J., et al.	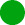	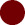	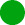	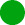	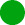	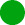	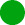	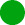	Low
Seo K. W., Yoon K. Y.	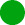	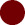	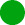	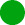	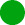	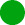	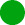	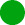	Low

Green = “yes”, red = “no”, and yellow = “unclear”.

**Table 3:** Summary results table.

Author	Year	Type of study	Type of procedure	Mortality	Hospital stay	Operative time (min)	Blood loss (mL)	Postsurgical complications	Age/Sex	Follow-up
Namikawa T., Maeda M., Yokota K., et al.	2021	Case report + review	Distal gastrectomy	None	12	335	20	None	74/M	mo
Sivakumar J., Crosthwaite G.	2021	Case report	Total gastrectomy	None	6	392	ND	None	29/M	h
Abbey E., Yang F., Qi L., et al.	2021	Case report	Robotic distal gastrectomy	None	15	205	20	None	69/M	mo
Takeno A., Masuzawa T., Katsuyama S., et al.	2021	Case report	Robot-assisted proximal gastrectomy	None	10	448	45	None	21/F	POD 10
Bawahab M. A.	2020	Case report	Laparoscopic sleeve gastrectomy	None	1	28	ND	None	84/M	mo
Yoshimoto T, Yoshikawa K, Tokunaga T, et al.	2020	Case report	Robot-assisted total gastrectomy	None	13	ND	30	None	80/F	POD 13
Ojima T., Nakamura M., Nakamori M., Yamaue H.	2019	Case report	Robotic distal gastrectomy	None	14	260	20	None	79/M	mo
Shibata K., Kawamura H., Ichikawa N., et al.	2018	Case report	Total gastrectomy	None	10	232	110	None	74/M	POD 10
Zhou, Lan et al.	2018	Case report	Subtotal gastrectomy	None	25	ND	ND	Bilateral pleural and pericardial effusion POD 3. ICU, intubation for respiratory failure POD 5	53/M	mo
Dai H. bin, Wang Z. C., Feng X. B., et al.	2018	Case report	Robotic radical gastrectomy	None	5	180	50	None	59/F	2.5 y
Villalvazo Y., Jensen C. M.	2018	Case report	Laparoscopic sleeve gastrectomy	None	3	108	ND	None	72/F	mo
Gundes, Cetin et al.	2018	Case study and review	Distal subtotal gastrectomy	None	8	150	100	None	50/M	—
Suh B. J.	2017	Case report	Robotic radical subtotal gastrectomy	None	10	180	ND	None	60/M	mo
Cao Y., Li J., Shen L., et al.	2017	Case report	Robotic total gastrectomy	None	8	ND	ND	None	54/F	d
Aziret, Karaman et al.	2017	Case report and brief review	Laparoscopic sleeve gastrectomy	None	5	105	ND	None	52/F	mo
Alhossaini R., Hyung W. J.	2017	Case report	Robotic-assisted distal gastrectomy	None	5	195	30	None	40s/M	mo
Kigasawa Y., Takeuchi H., Kawakubo H., et al.	2017	Case report	Laparoscopy-assisted distal gastrectomy	None	ND	284	40	None	21/F	y
Yazar F. M, Emre A., Akbulut S., et al.	2016	Case report and brief review	Laparoscopic sleeve gastrectomy	None	6	78	ND	None	58/M	8 mo
Morimoto M., Hayakawa T., Kitagami H., et al.	2015	Case report	Total gastrectomy	None	7	349	90	None	60/F	4 y
Ye M. F., Tao F., Xu G. G., Sun A. J.	2015	Case report	Distal gastrectomy	None	8	230	50	None	52/F	2 y
Genser, Tayar et al.	2015	Video report	Laparoscopic sleeve gastrectomy	None	ND	45	ND	None	66/F	—
Zhu H., Yang K., Hu J. K.	2015	Case report and review	Distal gastrectomy	None	ND	ND	ND	None	51/F	15 mo
Stier C., El-sayes I., Theodoridou S.	2014	Case report and brief review	Laparoscopic sleeve gastrectomy	None	5	61	ND	None	42/M	2 y
Sumi Y., Maehara R., Matsuda Y., et al.	2014	Case report	Distal gastrectomy	None	10	ND	ND	None	60/F	18 mo
Min S.-H., Lee C.-M., Jung H.-J., et al.	2013	Case report	Distal gastrectomy	None	Case 1–8 Case 2–5	Case 1–220 Case 2–117	Case 1–100 Case 2–50	None	52/M, 68/M	POD 8
Fujikawa H., Yoshikawa T., Aoyama T., et al.	2013	Case report	Laparoscopic distal gastrectomy	None	8 days after 2nd surgery	234	5	Mechanical obstruction due to adhesion requiring second operation	39/F	3 y
Deutsch G., Gunabushanam V., Mishra N., et al.	2012	Case report	Laparoscopic vertical sleeve gastrectomy	None	ND	ND	ND	Leak in gastrosplenic ligament adjacent to suture	47/M	2 y
Kim H. B , Lee J. H., Park D. J., et al.	2012	Case report	Robot distal gastrectomy	None	8	300	ND	None	60/M	15 mo
Seo K. W., Yoon K. Y.	2011	Case report	Laparoscopic distal gastrectomy	None	7	200	70	None	53/M	POD 7

**Table 4:** Types of gastrectomy procedures reported for SIT patients in the literature.

Type of gastrectomy reported	Subtype of gastrectomy	Number
Laparoscopic gastrectomy type	Distal	9
	Sleeve	7
	Total	3
	Subtotal	1
	Distal subtotal	1

Robot-assisted gastrectomy type	Distal	4
	Total	3
	Proximal	1
	Radical subtotal	1

**Table 5:** Comparison of mean operative time, intraoperative blood loss, age of the patients, and hospital stay duration for Laparoscopic and robot-assisted gastrectomy in SIT patients.

	Laparoscopic gastrectomy	Robot-assisted gastrectomy	Mean difference, 95% CI	*p* value
Mean operative time (min)	186.35 ± 111.62	252.57 ± 97.13	−66.22 (−154.09, 21.65)	0.1854
Mean blood loss (mL)	63.50 ± 36.21	32.50 ± 12.54	31 (5.43, 56.57)	0.0293
Mean age (yrs)	55.09 ± 15.16	58.00 ± 18.58	−2.91 (−16.14, 10.32)	0.6564
Mean hospital stay (days)	7.88 ± 5.14	9.77 ± 3.66	−1.89 (−5.77, 1.99)	0.3382

**Table 6:** Vascular anomalies found in SIT patients preoperatively.

Cases	Vascular anomaly
Namikawa et al.	CHA from SMA
Shibata et al.	Right gastroepiploic artery runs in front of the right gastroepiploic vein
Cao et al.	No CHA, HA derived from SMA, portal venous system superficial to celiac branches
Sumi et al.	LHA from SMA
Min et al.	Left gastric artery and CHA from SMA, right gastric artery from celiac trunk

^∗^CHA-common hepatic artery, SMA-superior mesenteric artery, HA-hepatic artery, and LHA-left hepatic artery.

## Data Availability

The data supporting this systematic review are from previously reported studies and datasets, which have been cited. The processed data are available from the corresponding author upon request.
